# Improving Multidisciplinary Team Working to Support Integrated Care for People with Frailty Amidst the COVID-19 Pandemic

**DOI:** 10.5334/ijic.7022

**Published:** 2023-06-05

**Authors:** Susan Barber, Michaela Otis, Geva Greenfield, Nasrin Razzaq, Deepa Solanki, John Norton, Sonia Richardson, Benedict W. J. Hayhoe

**Affiliations:** 1 Department of Primary Care and Public Health, School of Public Health, Imperial College London, The Reynolds Building, St Dunstan’s Road, London W6 8RP, UK; 2 Chelsea & Westminster Hospital NHS Foundation Trust, UK; 3 Harrow CCG The Heights, Middlesex 59-65 Lowlands Road Harrow HA1 3AW, UK; 4 Integrated Care Education, Harrow ICP and Training Hub, UK

**Keywords:** multi-disciplinary team, integrated care, workforce, training, collaborative learning, frailty, multimorbidity

## Abstract

Multidisciplinary team (MDT) working is essential to optimise and integrate services for people who are frail. MDTs require collaboration. Many health and social care professionals have not received formal training in collaborative working. This study investigated MDT training designed to help participants deliver integrated care for frail individuals during the Covid-19 pandemic.

Researchers utilised a semi-structured analytical framework to support observations of the training sessions and analyse the results of two surveys designed to assess the training process and its impact on participants knowledge and skills.

115 participants from 5 Primary Care Networks in London attended the training. Trainers utilised a video of a patient pathway, encouraged discussion of it, and demonstrated the use of evidence-based tools for patient needs assessment and care planning. Participants were encouraged to critique the patient pathway, reflect on their own experiences of planning and providing patient care.

38% of participants completed a pre-training survey, 47% a post-training survey. Significant improvement in knowledge and skills were reported including understanding roles in contributing to MDT working, confidence to speak in MDT meetings, using a range of evidence-based clinical tools for comprehensive assessment and care planning. Greater levels of autonomy, resilience, and support for MDT working were reported. Training proved effective; it could be scaled up and adopted to other settings.

## Introduction

Those who live with frailty and/or multimorbidity (MM) commonly require assistance from multiple health and social care services. Consequently, individuals may experience fragmented care and problems with healthcare access and navigation [1,2]. These problems may have been exacerbated by health services and societal changes relating to the Covid-19 pandemic, and lack of consistency in, and application of, formal education and training in multidisciplinary work [2,3,4,5]. Additionally, individuals with MM are at higher risk of Covid-19 infection [6], access to non-Covid-19 health and social care services was affected by a shift in resources to Covid-19-related activities, and those who were required to self-isolate experienced a reduction in support networks [2].

Whilst individuals with frailty have different care needs to those who live with MM, both require integrated care. A recent study of the coexistence of frailty and MM found an association: most (7/10) frail adults also had MM, whilst fewer (1/5) who have MM also have frailty [7].

Integrated care is intended to join up services around the needs of individuals, within or across community, primary or secondary health care settings, and social care [1,8,9,10], and has been a policy objective in England since 2012; [11,12] similar objectives exist in many other countries [13,14].

Multidisciplinary Team (MDT) working is a key component of system and service organisation for integrated care and has documented merits in facilitating positive outcomes for service users [[Bibr B1][Bibr B9][Bibr B8][Bibr B9][Bibr B15]]. In primary care, MDT collaboration that provides care for people with chronic health conditions including mental illness, shows greater improvements in outcomes than care by a single profession [15, p. 9]. Significant outcomes include reduced deterioration of health conditions and functional ability, reduced fear of falling, increased social activity, and improved activities in daily living, self-efficacy and the use of adaptive strategies [16].

Some attention has been paid by policymakers to provision of education and training to support MDTs to develop skills supporting provision of integrated care for people with frailty and/or who live with MM [17,18]. Few studies suggest how this could be operationalised during a pandemic [19]. Lack of staff training opportunities is one barrier to implementation of integrated care [1]. An observational study in the US showed that healthcare organisations were unsure of what skills and knowledge individuals required and underestimated the need for training. Finding clinicians with the skills and experience necessary to work in integrated care contexts proved difficult, and the time and resources needed to train clinicians and time for them to try out skills with MDT colleagues was underestimated. In organisations that provided training, it was uncommon for all individuals participating in the MDT to be offered it; it was recognised in retrospect that whole team training would have been more likely to achieve goals of implementation of integrated care [20, pp. 43–45].

### Study rationale

A consultation of service providers and patients in Harrow, Northwest London in 2019 highlighted sub-optimal MDT working as an obstacle to the provision of holistic patient care. The consultation suggested that integrated care for patients with, or at risk of, frailty required interprofessional training to prepare the workforce to manage frailty in an integrated manner [21]. Subsequent recognition of the additional contribution of the pandemic to problems with integrated care and care navigation strengthened the perceived need for this preparedness.

The purpose of this paper is to share learning from the implementation of training designed to strengthen the infrastructure required for one important aspect of successful integrated care, namely MDT working.

## The intervention

### Training for MDT working during the Covid-19 pandemic

Primary Care Networks (PCNs) within specific geographical locations in England exist to enable good quality personalised and integrated health services. Five cohorts of participants from 5 PCNs in Northwest London were trained between September and December 2020. Delivery of training focused on teams working in the same locality. Three sessions, lasting one hour each, were attended one month apart. Each covered a topic designed to improve skills and confidence for MDT working: i) multidisciplinary team culture, ii) clinical frailty toolkits, and iii) person-centred care planning.

Designed and delivered by facilitators with a background in clinical education, the training comprised formal and collaborative learning elements. Delivered over the Zoom platform, content and structure of the training provided opportunities for participants to develop a vision for MDT working during the pandemic; improve their knowledge of best-practice guidance; confidence to act as members of a collaborative team; understand their roles and the roles of others; become confident to participate in frailty assessment; plan and deliver care to frail people in the community, including in residential care homes.

Key elements of each training session were: practical demonstration of clinical tools; critique of a videoed case study of a patient journey; collaborative discussion about best use of clinical tools in relation to the case study, and reflective discussion.

Before and after each training session, participants were facilitated to communicate with each other through text-messaging platforms and videoconferencing. The establishment of practical networks to facilitate collaborative working after the training was encouraged.

#### Participants

Each training cohort comprised: GP, pharmacist, occupational therapist, physiotherapist, social prescriber, social worker, care-home manager, care-home health care assistant working within each PCN. Primary care managers invited 144 individuals to attend the training, selected on the perceived relevance of training to their ability to provide care during Covid-19.

### Evaluation of the intervention

The evaluation aimed to investigate how the training could help participants deliver integrated care for frail individuals during the Covid-19 pandemic and understand what impact the training had on participants’ confidence to act, their knowledge about other roles in the MDT and shared decision making. Desired learning outcomes, set by the training team, were used to structure the analysis of the impact of the training. They were informed by the frailty curriculum for health and social care professionals, which stipulated the need for training in the recognition, assessment, and collaborative management of frailty [21].

A sequential explanatory mixed-methods design [22,23] was used to assess how the training process achieved the learning outcomes. Mixed-methods sequential explanatory design allows for collecting and analysing quantitative data first, followed-up by qualitative data. This design allowed for cross-validation of findings from multiple sources of qualitative and quantitative data that were collected and analysed in sequence. Researchers used a semi-structured analytical framework informed by the desired learning outcomes to record observations of training sessions. This was cross-validated with quantitative analysis of pre-post participant surveys to evidence the impact of training. The learning outcomes were also used as a set of *a priori* themes to deductively explore participants’ qualitative survey responses about how learning outcomes were met through the training processes [24].

#### Qualitative strand: Researcher observations

Three researchers (two academics and one public representative) each observed three training sessions in two of the five PCN cohorts. Researchers recorded hand-written notes during and after the training, and utilised video-recordings of the sessions. Researchers recorded i) Process: reflections on the training structure, content, and activities; ii) Training delivery based on the trainer styles; and iii) Impact: the trainees’ behavioural responses to the training.

Researcher observations were analysed using deductive thematic analysis guided by an analytic framework incorporating the learning objectives. Observations were compared between researchers after each session, and points of disagreement were addressed by reviewing video-recordings of the sessions, to reduce observer bias.

#### A pre-post survey of training participants

A survey was sent by email, by training facilitators (NR and DS) before the first training session, and after the last training session. Qualitative and quantitative data were collected from two surveys. Participants were not asked to report identifiable information, which prevented matching participants’ scores between the pre- and post- training responses. However, 81.5% of respondents completed both pre- and post-training surveys. The 8.5% who completed the post-training survey only were selected from the same staffing cohort, thus, baseline scores were assumed to be comparable.

#### Quantitative strand: Survey

Survey variables were grouped under three learning outcomes [25]. Eleven pre-survey questions assessed perceived performance of the MDT, covering learning objectives 1) Establishing a team for shared decision making, 1a) contributions from all MDT members to facilitate shared decision-making, 1b) clarity about the team’s roles and individual responsibilities, and 2) Confidence using clinical tools for comprehensive assessment and care planning skills across clinical disciplines. The post-training survey asked an additional nine questions covering a third learning objective, 3) Effective networking and relationship dynamics, comprising twenty questions. All questions used a 5-point Likert response scale (ranging from 1 = “strongly disagree” to 5 = “strongly agree”, where 3 = “undecided”).

Analysis of the quantitative survey explored changes in the learning outcomes to assess the impact of the training. Quantitative survey responses were tabulated to display the difference between the pre- and post-training surveys, which were statistically compared to assess the change in learning outcomes after the training. Participants’ scores were not matched between pre- and post- surveys; thus, scores were dichotomised on whether participants reported that the learning outcomes were achieved (1 = ‘agree’ to ‘strongly agree’) or not (0 = ‘strongly disagree’ to ‘undecided’). Logistic regressions calculated odds ratios on the proportion of achievement between the pre- and post- surveys, where regression assumptions were met. To assess differences in the perceived impact of the training between clinical disciplines, Kruskal-Wallis comparison of ranks tests compared responses between i) general practitioners (GPs), ii) nurses, iii) pharmacists, iv) physiotherapists, occupational therapists and psychological therapists, v) social workers and social prescribers, vi) clinical consultants, and vii) managers, coordinators and administrators. Any disciplines with fewer than three respondents, or that could not be appropriately combined with other groups, were excluded from statistical comparisons to protect confidentiality.

#### Qualitative strand: Survey analysis

Participants were asked to share perceptions about whether the training had helped them to achieve each of the learning outcomes. Additional qualitative survey questions asked about challenges to or opportunities for MDT working during the Covid-19 pandemic in both surveys. The qualitative survey questions explored participants’ perceptions of how, and to what extent, the training processes achieved the desired learning outcomes.

### Cross-validation between qualitative and quantitative analyses

Researcher observations and survey responses were used to corroborate the impact of the training on achievement of the desired learning outcomes based on behavioural demonstration reported by the training attendees, and details of the training processes used to achieve it. Qualitative survey responses were used to corroborate evidence from the survey findings and provide detailed perspectives on the process of the training and whether desired learning outcomes were achieved. Respondents’ qualitative comments were summarised under each training objective.

## Results

We report three themes focussed on achieving the desired learning outcomes: 1) Establishing a team culture for shared decision-making, 2) Confidence using clinical tools for comprehensive assessment, and 3) Effective MDT networking and communication.

### Qualitative results – Observations of training

#### Theme 1: Establishing a team culture for shared decision making

Central to achieving shared decision making was to establish a positive team culture. The first training session utilised a method based on de Bono’s ‘Six Thinking Hats’ [26] designed to elicit participants’ reflections about the importance of a positive team culture. Facilitators encouraged reflection on the values of respect, gratitude, and recognition as building blocks for shared decision making, and holding each other to account for professional responsibility for safe care delivery. Feedback from one sub-group evidenced investment in team-based culture:

“[Our vision is for] honesty, transparency, clarity, and room for growth within the team… shared identity and purpose, seamless care… integrating on all levels – human, relational, technological – for information and knowledge sharing to allow people to be informed and empowered. Achieved through respect, and value for one another.”

Participants demonstrated competency to contribute to positive team culture by listening to each other during the group activity and sharing reflections on intentions to work collaboratively. The training format was authentic to the team-based model. Activities encouraged participants to contribute to the discussion and learn about each other’s clinical roles.

#### Theme 2: Confidence using clinical tools for comprehensive assessment

Conducting comprehensive frailty assessments required skills to identify and monitor frailty clinically, as well as the confidence for all MDT members to critique how and in what circumstances to use various standardised tools. To build confidence in comprehensive assessment, training facilitators discussed and demonstrated how to use a range of clinical tools designed for use in primary, secondary and care home settings to improve participants’ knowledge and skills for using these tools and practicing with them.

Completing a comprehensive assessment on a case example helped participants in sub-groups to understand the importance of multiple clinical disciplines:

“It was really useful to get into the mindset of other disciplines. [The fall that the patient had may have been due to] loss of confidence to go out during the pandemic – loss of strength from lack of exercise. We (the Ambulance Service) would contact the falls rehab to see if physiotherapy could attend the patient’s home, and occupational therapy to assess for hazards.”

Learning how to complete a comprehensive geriatric assessment using a patient case example was authentic to the learning outcome. This was achieved by dedicating a breakout group to each of the assessment domains: functional, psychological, physical, and pharmaceutical.

Tools to support assessment of end-of-life care needs enabled participants to act-out and share the sorts of important conversations that occur in their professional role, which demonstrated self-recognition of their integral role to the MDT and competency to contribute to the MDT discussion:

“People talk to me (a social prescriber)– sometimes they are depressed, sometimes suicidal, wanting something, not wanting something. I must make decisions about what to tell, and to whom, recognising the important things to pass on to [others].”

Training facilitators highlighted the value of seeking perspectives from those who know the patient in their everyday life, including non-clinicians. A role-play scenario demonstrated how to express concern for/about a patient in a way that adheres to the patient’s and carers’ opinions of the patient’s health, and to elicit their preferences for advanced care planning.

#### Theme 3: Effective MDT networking and communication

Working effectively as an MDT required all members to gain awareness of their own and others’ roles to appreciate the unique contribution of each discipline and position. With a greater appreciation of each individual contribution, the group reflected on the importance for channels of communication to work effectively as an MDT.

There were barriers to communication that were integral to the workplace systems. The group identified tendencies to work in silos that prevented cross-disciplinary communication. Participants expressed some hesitation in the ability to establish MDTs outside of the training:

“It’s difficult to communicate with other professionals for joined up care, because our IT systems don’t allow for it” (training participant: physiotherapist)

The training invited participants to reflect on ways to overcome barriers, through establishing MDT networking as a routine practice.

A group chat forum remained open before, during and after the training delivery. The chat was used for questions and answers, sharing examples of good practice, and reflections on applicability of learning to trainees’ professional roles.

The necessary elements of nurturing a ‘community of practice’ in action was discussed. Participants showed interest in setting up such a network to help support them after the training. Time constraints meant that this was deemed an issue to follow up after the training.

### Quantitative results – Participant survey

Of 144 staff invited to participate, 80% (n = 115) attended the training. Of 115 training attendees, 38% (n = 44) completed the pre-training survey in September 2020 at the start of the training, and 47% (n = 54) completed the post-training survey at the end of the training in December 2020. Ten participants (18.5%) completed the post-training survey only. As it was not recorded which participants did not complete the pre-training survey, all responses were included.

Participants who completed the survey comprised GPs (n = 20, 37%), nurses (n = 14, 26%), pharmacists (n = 8, 15%), social workers or social prescribers (n = 6, 11%), physiotherapists or occupational therapists (n = 5, 9%), and one administrator (1%). The administrator was omitted from analyses to preserve confidentiality.

The rate of agreement with each learning outcome is presented in Table 1.

**Table 1:** Pre-and post-training survey responses, and difference in agreement that MDT objectives were realised before and after training.


MDT OBJECTIVE	PRE-TRAINING SURVEY RESPONSES (N = 44)				POST-TRAINING SURVEY RESPONSES (N =54)				BEFORE TRAININGN =44	AFTER TRAININGN = 54	DIFFERENCE BETWEEN PRE- AND POST-TRAINING AGREEMENT

	DISAGREE	NEITHER	AGREE	STRONGLY AGREE	DISAGREE	NEITHER	AGREE	STRONGLY AGREE			

	N (%)				N (%)				AGREEMENT, N (%)		ODDS RATIO (95% CI)

Clarity in own role and responsibility	4 (9.1)	6 (13.5)	24 (54.7)	10 (22.7)	0 (0.0)	9 (16.5)	34 (63.1)	11 (20.4)	34 (77)	45 (83)	1.46 (0.72 to 2.97)

Clarity in roles and responsibilities of others	5 (11.3)	10 (22.7)	20 (45.5)	9 (20.5)	0 (0.0)	3 (5.4)	35 (64.8)	16 (29.8)	29 (66)	51 (95)	8.76 (3.40 to 22.60)*

Clarity in patient/carer role and responsibilities	5 (11.3)	15 (34.1)	18 (41.1)	6 (13.5)	0 (0.0)	8 (14.8)	31 (57.3)	15 (27.9)	24 (55)	46 (85)	4.74 (2.41 to 9.31)*

Confident to speak in MDT discussions	5 (11.3)	11 (25.0)	21 (47.6)	7 (16.1)	0 (0.0)	7 (13.0)	28 (52.0)	19 (35.0)	28 (64)	47 (87)	3.82 (1.87 to 7.78)*

Confident participating in care home round	14 (31.8)	10 (22.7)	15 (34.1)	5 (11.4)	4 (7.3)	18 (33.3)	25 (46.3)	7 (13.1)	20 (45)	32 (59)	1.75 (1.00 to 3.06)

Confidence in recognising clinical frailty	9 (20.6)	7 (15.9)	22 (50.0)	6 (13.5)	0 (0.0)	8 (14.8)	35 (64.8)	11 (20.4)	28 (64)	46 (85)	3.29 (1.85 to 5.84)*

Confident using structured frailty assessment	15 (34.1)	17 (38.6)	10 (22.7)	2 (4.55)	0 (0.0)	11 (20.4)	25 (46.3)	18 (33.3)	12 (27)	43 (80)	10.43 (5.88 to 18.49)*

Confident to manage patient with frailty	8 (18.2)	14 (31.8)	14 (31.8)	8 (18.2)	0 (0.0)	7 (13.0)	40 (74.1)	7 (13.0)	22 (50)	47 (87)	6.72 (3.32 to 13.61)*

Confident to initiate advanced care plans	16 (36.5)	10 (22.7)	12 (27.3)	6 (13.5)	3 (5.6)	18 (33.3)	21 (38.9)	12 (22.2)	18 (41)	33 (61)	2.27 (1.21 to 4.24)*

The MDT makes me feel more resilient	–	–	–	–	0 (0.0)	2 (3.7)	37 (68.5)	15 (27.8)	–	52 (96)	–

The MDT makes me feel a sense of belonging	–	–	-	–	0 (0.0)	11 (20.4)	31 (57.3)	12 (22.3)	–	43 (80)	–

The MDT makes me feel more supported	–	–	–	–	0 (0.0)	1 (1.9)	32 (59.3)	21 (38.9)	–	53 (98)	–

The MDT gives me more autonomy	–	–	–	–	0 (0.0)	8 (14.8)	32 (59.3)	14 (25.9)	–	46 (85)	–

I am more aware of my local colleagues	–	–	–	–	0 (0.0)	5 (9.3)	27 (50.0)	22 (40.7)	–	49 (91)	–

I am more aware of local patient services	–	–	–	–	3 (5.6)	10 (18.5)	33 (61.1)	8 (14.8)	–	41 (76)	–


CI = confidence interval, * = *P* < 0.05, ‘–’ indicates a missing response where data were not collected in the pre-training survey. Note: Disagree includes ‘strongly disagree’ responses, due to very few ‘strongly disagree’ responses that were reported.

#### (3.2.1) 1. Clarity of roles for shared decision-making

Before the training, some respondents did not understand their own (9%) and others’ (11%) roles in the MDT, nor felt confident speaking in in MDT discussions (11%). Most understood their own (77%) and others’ roles (66%) and felt confident contributing (64%). After the training, there was improved clarity of one’s own role (83%), others’ roles (95%), and confidence in contributing to MDT discussions (87%).There was a significantly improved understanding of the roles of others (OR = 8.76, 95%CI: 3.40–22.60), understanding of the role of patients and carers (OR = 4.74, 95%CI: 2.41–9.31), and confidence in speaking in MDT discussions (OR = 3.82, 95%CI: 1.87–7.78). Only 6% of the trainees noted an improvement in understanding their own role, rising from 77% before to 83% after training. Comparing disciplines, GPs, social workers and social prescribers expressed a lack of understanding in their own role in the MDT – around 15–30% in each clinical discipline before training. Yet, after the training, clinical disciplines had statistically similar levels of confidence in participating in MDT discussions and understanding other’s roles and responsibilities, except GPs and nurses of whom fewer expressed confidence, compared to all other disciplines (*H*(4) = 10.51, *P =* 0.03). In open-ended survey questions, four GPs reported embedding MDT working into their routine clinical practice after participating in the training programme.

#### (3.2.2) 2. Clinical tools for comprehensive assessment

Comparing the confidence rates before and after training, there was an improvement in the confidence for using structured risk assessments of clinical symptoms of frailty (27% vs 80%), initiating an advance care plan (41% vs 61%), conducting care planning (i.e., in care home rounds) (45% vs 59%), and managing patients with frailty (50% vs 87%).There was a significant improvement in confidence in recognising clinical frailty symptoms (OR = 3.29, 95%CI: 1.85–5.84), initiating an advance care planning discussion (OR = 2.27, 95%CI: 1.21–4.24), assessing frailty using a structured approach (OR = 10.43, 95%CI: 5.88–18.49), and managing a patient with frailty (OR = 6.72, 95%CI: 3.32–13.61). Only 6% of the cohort reported an improvement in their confidence to participate in care home rounds, which was non-significant, possibly due to the small sample size. Participants reflected on their ability to think from multiple perspectives after the training, to conduct comprehensive assessments.

“[learning to use] The clinical frailty scale has helped me recognise frailty and its different levels” (GP, post training survey)

“I will start using [comprehensive assessments] as springboards towards drawing in other MDT members” (Pharmacist, post-training survey)

“[the training has helped me to] think from the patient perspective” (Nurse, post training survey)

There were no differences in skillset scores between clinical disciplines, except for GPs and nurses, of whom fewer expressed confidence in recognising clinical frailty, compared to all other disciplines (*H*(4) = 16.19, *P <* 0.01).

GPs, nurses, and social workers/prescribers (n = 17) commented that they were planning to embed structured assessments and care planning tools into routine practice after participating in the training. Some participants expressed needing more practice on these tools to achieve MDT competency.

#### Effectiveness of MDT relationships

After participating in the training programme, many participants reported positive relationship building during the training. Being part of an MDT during training gave participants a greater sense of resilience (96%), support in their role (98%), autonomy (85%) and belonging (80%).

Participants valued the chance to build positive working relationships with other MDT members:

“It was a great opportunity to meet others in the MDT network and also to learn about how [patients with frailty can be supported]” (Social Prescriber/Worker)

“[I enjoyed] being able to interact with colleagues from other disciplines & locations which one does not usually get a chance to.” (GP)

“[I will] encourage my team to liaise with other professionals to achieve the best outcome for [patients].” (Manager/Co-ordinator)

Many commented that the training programme allowed them to meet other professionals in the network and learn about their work, improving the potential for cross-site support.

However, there were a small number of participants who did not experience positive relationships in their MDT; this did not significantly differ between disciplines. For instance, some expressed challenges relating to poor communication between MDT members, a lack of presence of senior clinicians and/or other key clinicians, or a view that individual perspectives are competing rather than complementary. This indicated that for some participants in the training there remained scepticism about siloed working relationships and the challenges involved in establishing change.

“The biggest challenge is communication between MDT members” (Nurse)

“I would have preferred to actually see senior clinicians from community care providers e.g. mental health team, community nursing, etc” (GP)

“People are still siloed by their professional or organisational perspective” (Consultant)

#### Challenges and opportunities of MDT working during Covid-19

Other challenges and opportunities reported in free-text questions described that caring for patients during the Covid-19 pandemic required adjustment to new technologies, changes in workload, and clinical Covid-19 guidelines.

After participating in the training, some participants expressed improved understanding of how to utilise virtual platforms to achieve MDT working yet recognised some limitations without in-person communication and IT interoperability between primary and secondary care. Participants recognised that using virtual platforms allowed more staff to attend the meeting than in person, which improved the capacity for multidisciplinary assessment, timeliness of care provision, care continuity, and comprehensive care coordination.

The incompatibility between IT systems in primary and secondary care was described as time consuming and frustrating, especially for staff using multiple systems. This prevented cross-site sharing of information within the MDT. Working remotely was challenging because of limitations in accessing IT systems using a remote connection due to poor internet connectivity. Virtual meetings reduced the ability to build rapport with patients and between MDT members – particularly for accurately assessing patients relating to reduced visibility for nonverbal communication.

Some respondents worried about time constraints and being able to commit to future MDT meetings, due to increased demand and workload because of the pandemic and adjusting to frequent changes to clinical guidelines, plus difficulty maintaining MDTs when staff often held part-time employment contracts.

## Discussion

### Main findings

Training supported members of MDTs to deliver integrated care for patients during the Covid-19 pandemic. The setting, methods and materials utilised were appropriate to supporting teams who work in the same locality develop knowledge and confidence to act. Opportunity to critique a patient journey was well received, provided a focus for dialogue and exchange of experiential knowledge, and promoted shared decision making.

Improvement in knowledge and skills such as individuals’ recognition of their own role, those of others in the MDT, including patients’, was reported. Confidence to speak in MDT meetings, to use a range of clinical assessment tools for comprehensive assessment and care planning, increased autonomy, resilience, and support for MDT working were also reported as was the recognition of the role that these factors play in building and maintaining mutual respect and trust. Training helped participants navigate practical needs associated with the pandemic, such as: the use of virtual platforms, adhering to guidelines on social distancing; learning how to prioritise necessary face-to-face interactions.

### Interpretation

The evaluation is innovative in considering MDT workforce training as a key aspect of integrated care. This is recognised in studies from around the world as necessary, and as a challenge. Below, we discuss the issues involved in achieving robust MDT working and consider the challenge that insufficient workforce culture change is partly responsible for lack of delivery of consistent integrated care.

Hallmarks of effective MDTs that support integrated care are: excellent team culture, communication and mutual trust between team members, establishment of mutual understanding of patient’s needs and subsequent actions to be taken [1,9,15,16]. This study demonstrated how these qualities and skills can be nurtured in training and reflective learning.

Team culture influences the likely outcomes of MDT interventions. Factors influencing the success of interventions include the degree of cooperation, shared team identity, shared competencies, setting and working towards common goals, and shared decision making [9], [16]. Participants in this case study were encouraged to critique the care received by the patient in the video used during training sessions and reflect on what they would have done differently in light of what they had learned. Shared competencies and establishment of common goals were encouraged through utilising standardised protocols such as geriatric assessment to guide diagnosis, therapies and treatments [16].

Enhanced team performance is associated with close working relationships, the joint identification of the needs of patients, and knowledge sharing [9]. Positive patient and service provider outcomes have been associated with MDTs that establish trusting interprofessional relationships, communication methods to support a comprehensive understanding of and commitment to addressing the needs and perspectives of service users [15,16]. These factors were discussed during the training and challenges to securing the necessary improvements to interprofessional relationships were raised, for example the need to make time to collaborate, and for an integrated IT system to facilitate integrated care. Participants were encouraged to test solutions and/or raise issues in their MDTs to try to improve these in between each of the training sessions.

Honest and open communication between team members, and with patients families and carers, are considered crucial to team building, and to valuing participants’ knowledge and experience. Confidence to build robust MDT culture relies on timely and optimal contribution to MDT by all who provide care for a patient [16, pp. 105–107] as is the standardisation of skills and competencies needed to deliver integrated care [20]. This was encouraged throughout the training, especially through the critique of the video-based patient case study, with participants collaborating as if they were working in a ‘live’ team context. In addition, teams were trained together with staff members working in the same locality. This contrasts positively with the experience of some teams that aimed to integrate care and who struggled to standardise their approach to doing so described by Hall et al [20]. Hall’s study described staff from mental health and primary care settings who experienced lack of shared understanding about how to assess and improve integrated care.

Training can play a role in supporting MDTs deliver integrated care. When this is backed up by effective inter-professional networking, practical interactions can be further nurtured [27,28]. Encouragement of networking, reported in this study could be a crucial next step to developing and implementing integrated care, in England, and internationally.

### Implications for research

The research reported on above could be followed up to examine the sustainability of the impact observed, the use made of the training by the participants, and the impact on patient-care. Testing the model with a larger cohort of participants, in different contexts; extending the scope of the training to include support for networking, may help bring about culture change to help deliver integrated care.

To conduct robust evaluation of training for integrated care it is necessary to monitor the impact on delivery of integrated care. Thus, pre-post designs should include before-after comparison of integrated care indicators at the study sites, as well as patient-reported outcomes on integrated care.

Ideally patients and carers would have been included in the training directly, bringing their own experience to share. Future research should include assessment of integrated care including patient experience and patient-reported quality of care.

A variety of tools exist to aid assessment of frailty [29,30,31]. Most frail adults also have MM, whilst fewer who have MM also have frailty [7]. The developers of the training observed in our study were aware of the above and, for the purpose of pursuing the learning objective of creating shared knowledge and confidence to act according to an evidence-based tool, they chose to use the Comprehensive Geriatric Assessment in the training, and they emphasised the need for the MDT to have common frameworks. In line with Vetrano et al [7] more research is needed to provide rigorous and standardized measurements of both MM and for both clinical and research purposes.

### Implications for practice and policy

Workforce readiness to provide integrated care, and to work in effective MDTs is not comprehensively practised. A recent evaluation of Integrated Care Pilot Projects (ICPs) in England suggested that effective senior leadership, shared values, simple interventions, and the availability of additional funding supported successful efforts. It also found evidence of poor professional engagement, little shared understanding of the purpose or vision for integrated care, and challenges pertaining to information and data-sharing [10].

Support for professional development in MDT settings is needed. Mobilising behind this aim could prove crucial to the success of efforts raised in policy and legislation promoting MDT working with necessary training to make it effective [11,12,13,14], [32,33,34]. More focus on training and education for MDTs and the training of teams is required [4,5,35,36] including in countries where training opportunities are scarce and care for frail patients is minimal or absent [36]. The value of nurturing a ‘community of practice’ has been outlined by others [27,28] but this has not, to our knowledge been applied to improving MDTs working with people with frailty or MM. Taking a hybrid approach to training and building a community of practice could have been useful to add to the training that we observed. Additionally, training for multidisciplinary care working to improve integrated care would benefit from the inclusion of patients and carers in the planning and execution of training sessions, as either participants and/or as experts with lived experience.

There is a need to resource and implement fully interoperable IT to facilitate the use of comprehensive patient records across primary and secondary care settings to enable service providers and patients access and to support MDT working.

Learning from this study could be adopted or adapted to new geographical contexts. The model described in this study could be used inform approaches to scaling up capacity and developing culture change designed to promote MDT skills and habits in the workforce [37], [38]. It suggests that a model using evidence-based guidelines to promote shared knowledge and confidence to contribute to MDT working should be an integral part of that effort, that the development of practical networks need to be engineered and these could be an important part of the necessary workforce development required to promote integrated patient care. More testing is required in different contexts, along with evaluation. This could help facilitate normalisation of MDT interactions whether these are in virtual, or face to face settings.

## Lessons Learned

Key lessons are:

- Training crafted to the needs of local MDT staff, involving patients and carers in the design of it and promoting collaborative learning, with a focus on a specific service user cohort, is likely to improve knowledge and confidence to act among staff who must organise and deliver integrated care.
- Infrastructure to support training, promote knowledge acquisition and shared decision making, practical mentoring, and the development and use of communities of practice are likely to be required to deliver integrated care.
- The model reported in this study is likely to be adaptable to other geographic settings.
- MDT skills may be built into education and day-to-day practice, including in countries where frailty services and training are minimal or absent.
- Focus on what matters most to service users is essential to achieving best outcomes in care planning and delivery for frail people, including towards the end of life.

## Strengths and limitations

### Strengths

The intervention focused on a crucial aspect in the infrastructure necessary to deliver integrated care, namely the provision of training for and collaborative learning among MDT members. The study evaluated the intervention using mixed-methods: key literature review, survey, observations. Survey responses were collected promptly before and after training, facilitating participants to indicate their level of skill, knowledge, and confidence to act, before and after the training. This was likely to improve the validity of responses.

### Limitations

The intervention was implemented as a response to the pandemic and not holistically connected to wider integrated care infrastructure. Whilst there is no clear agreement about the infrastructure required, it is likely to include the development of a broad inter professional education and training infrastructure; subsequently maintaining MDT knowledge, skills, and integrity in shared decision-making and patient-centred service delivery. No patients or carers were invited to the training, and this is a weakness in the training methods observed. We did not have access to patient data from the study sites, thus, it was not possible to assess the impact of training on delivery of integrated care.

Participant scores were not matched between pre-and post-training surveys. Instead, the before and after agreement rate of whether MDT objectives were realised were compared using logistic regression, rather than comparing changes in individual scores. Thus, it was not possible to exclude data from late joiners or run sensitivity analyses. Due to the small sample size, some clinical disciplines were amalgamated, yet individuals from different disciplines were likely to have different training response styles. There may have been confounders on the improvement in learning outcomes, such as participants’ years of professional experience; however, these were not measured, and their effects are unknown.

The training sessions took place on Zoom which meant that individuals were only visible on screen when then spoke. Observations of emotions, interactions, body-language that are possible in face-to-face training were difficult in this environment. For example, it was not possible to elicit observational evidence on issues such as barriers in communication between participants and working in silos. Given these limitations on interpreting observations of the training, we adopted an explanatory stance, evidencing how training was effective, for whom, using available data.

Potential sources of bias were i) selective recruitment of participants deemed appropriate by primary care managers to benefit from the training, ii) the attrition of participants who dropped out of training. Both might have conflated the observed improvement in outcomes. To counteract these biases, the corroboration between researcher observations of the achieved learning outcomes with participant surveys helped to identify perspectives that were shared by both observers and participants.

## Conclusion

Appropriate training for MDT working is essential and should be a prerequisite to effective use of MDTs. Ongoing reflective practice, possibly supported by an external facilitator, could help support constructive team dynamics. Whilst policy has been in place for some time, MDTs are innovative, and many health and social care workers have not received formal training in collaborative work. MDTs introduce a model where collaboration is needed across professions and ideally everybody’s voice is equal. Sharing learning about each other and the priorities and perspectives of other professionals can make multidisciplinary training effective for the benefit of staff and patients. As one participant in the training put it: *“It was really useful to get into the mindset of other disciplines”*.

This study provides an example of an intervention that focused on the delivery and outcomes of training to develop the skills and confidence required for successful integrated care for patients with frailty, and it fills a gap in knowledge about how to approach this.

Whilst the intervention took place during the Covid 19 pandemic and was focused on how to establish and improve MDT working to achieve this, insights have relevance in non-pandemic contexts and for locations outside of the UK.

## Disclaimer

This article presents independent research supported by the National Institute for Health Research (NIHR) under the Applied Health Research (ARC) programme for Northwest London. The views expressed in this publication are those of the author(s) and not necessarily those of the NHS, the NIHR or the Department of Health and Social Care.

## Obituary

Co-author, John Norton, passed away on 3 April 2023 at the age of 96 years. The co-authors would like to express our sadness at his death and highlight his contribution to this and the NHS/research community more broadly. John was an advocate for patient and public collaboration in research. His contributions will be sorely missed, but his legacy will live on through the lasting impact he has had on the health sector and the lives he touched.

## Additional File

The additional file for this article can be found as follows:

10.5334/ijic.7022.s1 Measurements of study of the variables.
